# Supplementary material for: Ultra-fast deep-learned CNS tumour classification during surgery
Source: Nature. 2023 Oct 11;622(7984):842–9. doi: 10.1038/s41586-023-06615-2 (PMC10600004; doi:10.1038/s41586-023-06615-2)
Supplement: Supplementary file 1 — This file contains the Supplementary Note, Supplementary Figures 1–24, a link to Supplementary Video 1 and legends for Supplementary Tables 1–10. [file 41586_2023_6615_MOESM1_ESM.docx]

**Ultra-fast deep-learned CNS tumor classification during surgery**

*Supplementary data file*

C. Vermeulen^1,2†^, M. Pagès-Gallego^1,2†^, L. Kester^3^, M.E.G. Kranendonk^3^, P. Wesseling^3,4^, N. Verburg^5^, P. de Witt Hamer^5^, E.J. Kooi^4^, L. Dankmeijer^4,5^, J. van der Lugt^3^, K. van Baarsen^3^, E.W. Hoving^3^, B.B.J. Tops^3*^ and J. de Ridder^1,2*^

^1^Oncode Institute, The Netherlands.

^2^Center for Molecular Medicine, UMC Utrecht, Heidelberglaan 100, Utrecht, The Netherlands.

^3^Princess Máxima Center for Pediatric Oncology, Utrecht, The Netherlands.

^4^Department of Pathology, Amsterdam University Medical Centers/VUmc, Amsterdam, The Netherlands.

^5^Department of Neurosurgery, Amsterdam University Medical Centers/VUmc, Amsterdam, The Netherlands.

^*^Corresponding author(s). E-mail(s):

b.b.j.tops@prinsesmaximacentrum.nl; j.deridder4@umcutrecht.nl;

^†^These authors contributed equally to this work.

**Contents**

**Supplementary Note:** Location specific classification

**Supplementary Video 1** legend

**Supplementary Table** legends

**Supplementary Figure 1:** Concordance between nanopore sequencing and Infinium arrays. **Supplementary Figure 2:** F1 scores for each class at different simulated sequencing depths. **Supplementary Figure 3:** F1 scores on the family level at different sequencing depths.

**Supplementary Figure 4:** Expected versus observed True Positive Rate for each different class in the validation fold prior to calibration.

**Supplementary Figure 5:** Expected versus expected True Positive Rate for each different class in the validation fold after temperature scaling.

**Supplementary Figure 6:** Expected versus expected True Positive Rate for each different class in the test fold prior to calibration.

**Supplementary Figure 7:** Expected versus expected True Positive Rate for each different class in the test fold after temperature scaling.

**Supplementary Figure 8:** True positive rate for each class when using a cutoff of 0.8. **Supplementary Figure 9:** Confusion matrix for pediatric samples using a cutoff of 0.95. **Supplementary Figure 10:** Confusion matrix for pediatric samples using a cutoff of 0.8. **Supplementary Figure 11:** Overlap between the nanoDx pipeline and Sturgeon classification on an external dataset.

**Supplementary Figure 12:** MinION sequencing metrics.

**Supplementary Figure 13:** Classification results from a retrospective oligodendroglioma case (UMCU_1).

**Supplementary Figure 14:** Copy Number Variation profiles for PMC_60.

**Supplementary Figure 15:** Copy number alterations for four nanopore sequenced samples com- pared to whole exome sequencing based Copy number alterations.

**Supplementary Figure 16:** Brainstem classifier confusion matrix and F1 scores. **Supplementary Figure 17:** Confidence over time for the brainstem classifier on brainstem samples.

**Supplementary Figure 18:** Confidence over time for the general classifier on brainstem sam- ples.

**Supplementary Figure 19:** Confidence over time for the brainstem classifier on non-brainstem samples.

**Supplementary Figure 20:** Robustness of the brainstem and general classifier for brain- stem samples.

**Supplementary Figure 21:** Robustness of the brainstem and general classifier for non-brain- stem samples.

**Supplementary Figure 22:** Read length adaptive versus non adaptive sampling. **Supplementary Figure 23:** Throughput of adaptive versus non adaptive channels. **Supplementary Figure 24:** Robustness of adaptive versus regular sequencing.

**Supplementary note: Location specific classification**

The Capper *et al*. dataset encompasses 81 tumor classes. However, many class distinctions are only relevant within a particular topological context, and could therefore already be ruled out prior to surgery. For instance, for a surgery of the spinal mass, a classifier does not need to be able to detect pituitary adenomas. Compared to other regions in the brain, the number of relevant classes in the brainstem is relatively low (N=21, **Supplementary Table 9**). We hypothesized that, by merging the irrelevant classes from the training dataset into a single class, the model can focus on the truly relevant classes and improve its performance. To test this hypothesis, we developed a Sturgeon classifier specifically for brainstem tumors. We trained the model on the complete Capper et al. dataset, however, non-brainstem classes were grouped together as a single class (“Other - Non Brainstem”) with the exception of the control classes (**Supplementary Fig. 16**).

We first evaluated this brainstem-specific classifier on the 94 pediatric EPIC profiles, to allow comparison with the general Sturgeon classifier results. On samples with a high Heidelberg confidence score for which the class was included in the training data (N=56), the brainstem-specific classifier performance was slightly better at early time points than the general classifier (91% accurate classifications and 0.6% misdiagnoses at timepoint 1). As expected, performance is poor in samples that could not be definitively classified based on the Heidelberg classifier (55% correctly diagnosed at timepoint 1). When applying the brainstem classifier to samples from classes that do not typically occur in the brainstem (N=23), Sturgeon does not misclassify, but rather classifies the sample as unclear (‘Other - Non Brainstem’ or low confidence score) in the vast majority of cases (**Supplementary Table 3**).

To further evaluate the performance of the brainstem-specific classifier, we obtained 24 additional tumor DNA samples from the PMC biobank that originated in the brainstem (two of which; PMC_42 and PMC_105 were also included in the previous analyses). Reflective of the epidemiology of pediatric brain- stem tumors, the vast majority of samples (23 out of 24) were diagnosed with a Glioblastoma harboring an H3K27 alteration. We multiplex-sequenced these samples in two batches on the PromethION platform to a depth of >100,000 reads per sample.

We then evaluated the performance of both the brainstem-specific and general classifier on an increasing number of covered CpG sites, reflecting the coverage expected in a single-sample MinION run (**Fig. 5a**) (**See Methods section Pseudotime**). No confident diagnosis (confidence score >0.95) was reached in 3 out of 24 samples by the general classifier; in those cases, the brainstem classifier did not succeed either or confidently classified them as “Other - Non Brainstem” (PMC_116, PMC_123 and PMC_127) (**Supplementary Fig. 17, 18**). Notably, in three out of five cases where the EPIC array classification was unclear (score below 0.84), a diagnosis was reached by both classifiers (PMC_114, PMC_121, PMC_122). In cases where both models reached the 0.95 threshold, the brainstem classifier was faster in 10 cases (saving, on average, 5 minutes of sequencing).

Finally, we tested the brainstem classifier on non-brainstem samples. The brainstem classifier correctly classified samples whose class is included in the brainstem classes, while classifying the rest as non-brainstem (**Supplementary Fig. 19**). We further assessed the added value of the brainstem classifier by generating 100 random read samplings per time point and comparing the scores between the brainstem and the general classifier. Overall, the brainstem classifier achieves the 0.95 threshold earlier (10 minutes on average) than the general classifier. (**Supplementary Fig. 20**). A similar trend can also be observed on samples that are not from the brainstem, but that fall within the brainstem classes, with the exception of 3 samples (**Supplementary Fig. 21**). Detailed results are listed in Supplementary Table 10. To mitigate the risk of a misdiagnosis due to overfitting, or when an unusual tumor type is encountered in the brainstem, and to take advantage of both classifiers, the brainstem classifier can easily be deployed in parallel to the general Sturgeon classifier.

**Supplementary Video 1:** An intraoperative sequencing experiment (INTRA_4) was captured on film. Audio has been redacted for privacy reasons. The timeline of this specific experiment is shown as a representative example in Fig. 4. The video is available for download via <https://zenodo.org/record/8261128>, DOI: 10.5281/zenodo.8261128.

*File: Supplementary_Table_1.xlsx*

**Supplementary Table 1:** Methylation calling concordance between Infinium arrays and nano- pore sequencing. Five samples were sequenced with different chemistry versions to benchmark the analysis pipelines. For each sample a 450K array was available. Sample: Sample name. Chemistry: The flowcell and library prep version used. window: The CpG calling window used to generate CpG methylation calls, majority voting was used for all CpG sites within the window, centered on the 450K target CpG site. Sites with evenly split votes were discarded. Concordant: The number of nanopore sequenced sites with a call identical to the (binarized) Infinium array call. Discordant: The number of nanopore-based calls in disagreement with the binarized Infinium array call. Total: The total number of CpG sites interrogated by both the methylation array and nanopore sequencing. Concordant (%): The percentage of concordant sites out of the total covered sites. Discordant (%): The percentage of discordant sites out of the total.

*File: Supplementary_Table_2.xlsx*

**Supplementary Table 2:** Expected Calibration Error per class on the validation and test folds, before and after calibration. Calibration was performed using temperature scaling tuned on the validation folds. This table lists the effect of calibration per class on the validation fold and on the test fold per class label.

*File: Supplementary_Table_3.xlsx*

**Supplementary Table 3:** Sturgeon performance on a pediatric CNS tumor patient cohort. 94 EPIC profiles from a pediatric cohort, were collected. For each sample we indicate the clinical diagnosis if available. We also translated the clinical diagnosis into the closest class within the 2018 Capper et al. system if applicable. Epic arrays were submitted to the Heidelberg classifier (V11b4 unless other- wise indicated). For each profile, 500 sequence experiments at each of the seven different timepoints were simulated and the Sturgeon classifier was applied. Results are listed for timepoints 1, 3 and 6. At each timepoint we indicate the number of correct outcomes at a cutoff of 0.95 (first table) or 0.8 (second table), the number of unclear outcomes, meaning the score was below the cutoff or a control class reached the threshold. Finally, we also indicate the number of wrong outcomes where an incorrect class reached the threshold. Next, we indicate the most frequently found unclear or wrong class at each timepoint. In the last two tables we show results for the brainstem classifier, here samples are split based on whether their class is included in the classifier scheme.

*File: Supplementary_Table_4.xlsx*

**Supplementary Table 4:** Tumor fractions in a series of intraoperative frozen section diagnoses. Year: year of sampling. Tumor cell percentage: The tumor fraction as estimated by the pathologist analyzing the intraoperative frozen section. Intraoperative Histology Diagnosis: The provisional diagnosis at the time of surgery. Patient nr. The patient identifier for samples where the material was also biobanked and used for the present study. Material used for molecular analysis: Whether the material used for frozen section diagnosis was also used for molecular diagnosis. Sturgeon result: The sturgeon classification for samples where the material was also used in the present study.

*File: Supplementary_Table_5.xlsx*

**Supplementary Table 5:** Retrospective nanopore sequencing runs. Reads were re-sampled in the order they were sequenced at a rate corresponding to an average minION sequencing run. Each time- point corresponds to approximately 5 minutes of sequencing. At each timepoint the number of covered 450K CpG sites is indicated, as well as the highest confidence score, the highest scoring class and the class that corresponds to the integrated histomolecular diagnosis. Finally, the score for each of the classes is shown.

*File: Supplementary_Table_6.xlsx*

**Supplementary Table 6:** Robustness analysis for retrospectively sequenced samples. 100 nanopore sequencing runs were simulated at different timepoints (0 - 11). Table lists the minimum, maximum and mean number of 450K CpG sites covered at each timepoint. The outcomes of running sturgeon on the 100 simulations are indicated, listing the number of incorrect outcomes above and below the 0.95 confidence threshold and the number of correct classifications below and above the

0.95 confidence threshold.

*File: Supplementary_Table_7.xlsx*

**Supplementary Table 7:** Performance on an external dataset. Sturgeon was applied to each sample; scores and outcomes were compared between the nanoDX pipeline and Sturgeon.

*File: Supplementary_Table_8.xlsx*

**Supplementary Table 8**: Results of 25 intraoperative samples. Reads were re-sampled in the order they were sequenced at a rate corresponding to an average minION sequencing run. Each timepoint corresponds to approximately 5 minutes of sequencing. At each timepoint the number of covered 450K CpG sites is indicated, as well as the highest confidence score, the highest scoring class and the class that corresponds to the integrated histomolecular diagnosis. Finally, the score for each of the classes is shown.

*File: Supplementary_Table_9.xlsx*

**Supplementary Table 9:** Classes included in the brainstem classifier.

*File: Supplementary_Table_10.xlsx*

**Supplementary Table 10:** Results of the brainstem classifier. Reads were re-sampled in the order they were sequenced at a rate corresponding to an average minION sequencing run. Both the brain- stem and general model are applied to the same set of reads. Each timepoint corresponds to approximately 5 minutes of sequencing. At each timepoint the number of covered 450K CpG sites is indicated, as well as the highest confidence score, the highest scoring class and the class that corresponds to the integrated histomolecular diagnosis. Finally, the score for each of the classes is shown.

**Supplementary Figure 1:** Concordance between nanopore sequencing and Infinium arrays. a. Illumina Infinium arrays of five samples were binarized using different beta cutoffs (x-axis) and the calls per CpG site were compared to those generated using nanopore sequencing (R9 chemistry, promethION flowcell, Megalodon methylation calling). b. The same data was compared using a symmetrical two-sided cutoff (sites with a beta value in-between the cutoffs were discarded). c. The fraction of interpretable sites when using two sided cutoffs and discarding sites with beta values in-between the cutoffs.


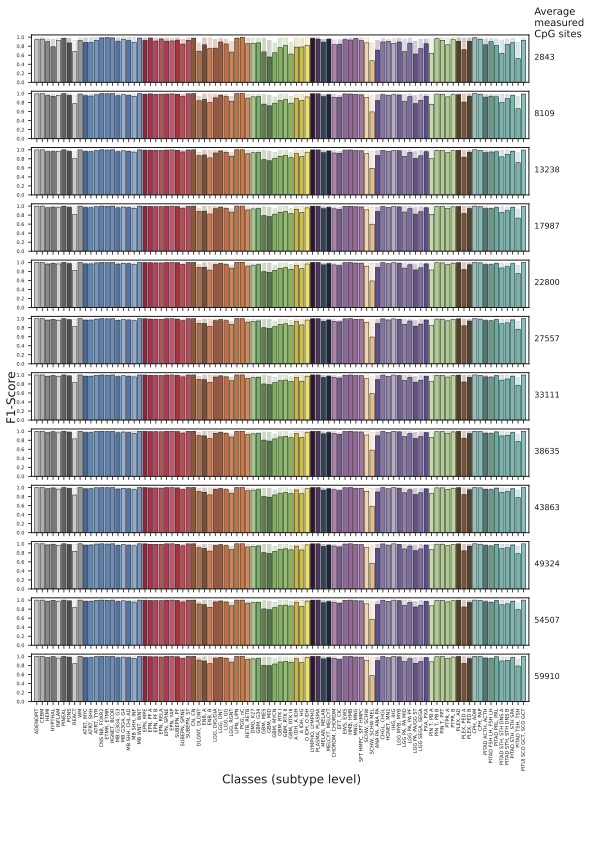


**Supplementary Figure 2:** F1 scores for each class at different simulated sequencing depths.

Transparent bars indicate performance when taking the top 3 scoring classes into account.

**Supplementary Figure 3:** F1 scores on the family level at different sequencing depths.

**Supplementary Figure 4:** Expected versus observed True Positive Rate for each different class in the validation fold prior to calibration. Red bars highlight deviation between expected and true TPR. Bottom right plot represents all aggregated classes.

**Supplementary Figure 5:** Expected versus expected True Positive Rate for each different class in the validation fold after temperature scaling. Red bars highlight deviation between expected and true TPR. Bottom right plot represents all aggregated classes.

**Supplementary Figure 6:** Expected versus expected True Positive Rate for each different class in the test fold prior to calibration. Red bars highlight deviation between expected and true TPR. Bottom right plot represents all aggregated classes.

**Supplementary Figure 7:** Expected versus expected True Positive Rate for each different class in the test fold after temperature scaling. Red bars highlight deviation between expected and true TPR. Bottom right plot represents all aggregated classes.

**Supplementary Figure 8:** True Positive Rate for each class when using a cutoff of 0.8. Asterisks indicate samples where the TPR is below 0.8.

**Supplementary Figure 9:** Confusion matrix for pediatric samples using a cutoff of 0.95. For each sample 500 nanopore runs were simulated at timepoint 1 and 3. The number of Sturgeon outcomes for each class is indicated in greyscale, unclear outcomes are also listed in the bottom row. Red squares indicate the Heidelberg classifier outcome (if conclusive), the blue cross indicates the clinical diagnosis.

**Supplementary Figure 10:** Confusion matrix for pediatric samples using a cutoff of 0.8. For each sample 500 nanopore runs were simulated at timepoint 1 and 3. The number of Sturgeon outcomes for each class is indicated in greyscale, unclear outcomes are also listed in the bottom row. Red squares indicate the Heidelberg classifier outcome (if conclusive), the blue cross indicates the clinical diagnosis.

**
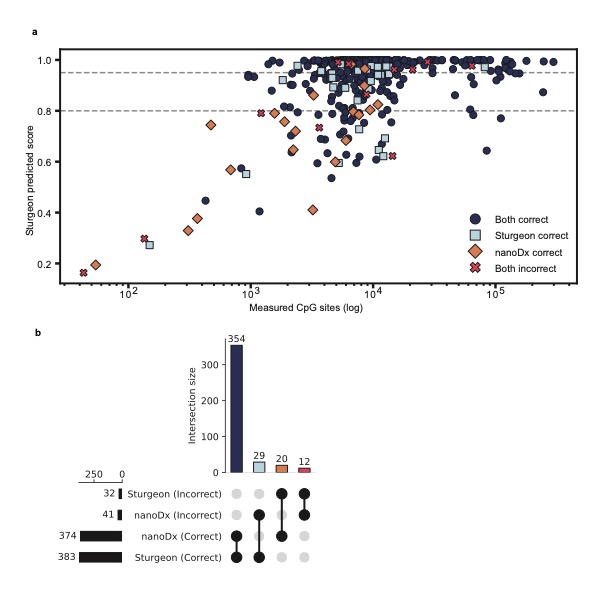
Supplementary Figure 11:** Overlap between the nanoDx pipeline and Sturgeon classification on an external dataset. a. y-axis indicates the sturgeon confidence score for each sample. X-axis indicates the number of measured (450K array) CpG sites. Samples are colored by the performance of both Sturgeon and nanoDx. Dark blue indicates that both methods were correct (N=354), light blue indicates that nanoDx was incorrect and Sturgeon was correct (N=29). Orange indicates that nanoDX was correct and sturgeon was incorrect (N=20), red indicates both methods were incorrect. b. Upset plot showing the performance of sturgeon and nanoDX.


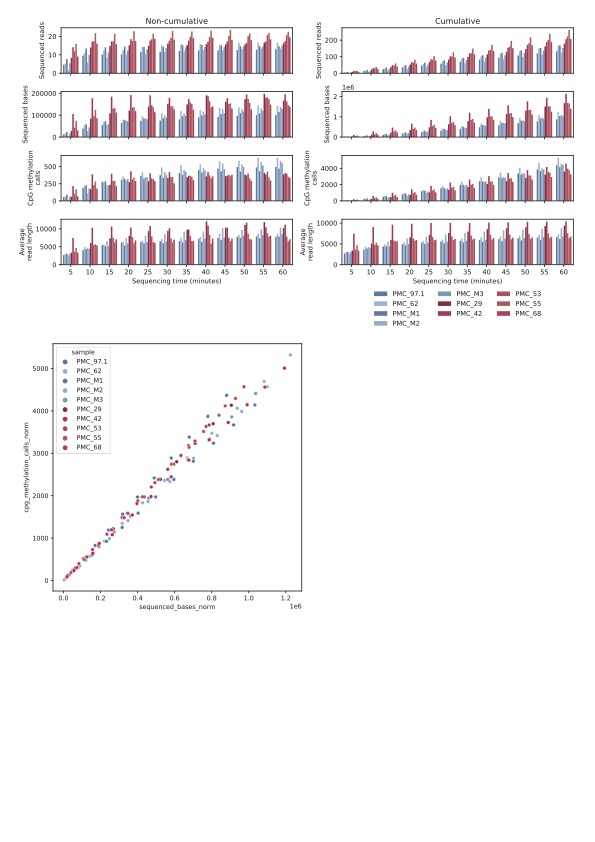


**Supplementary Figure 12:** MinION sequencing metrics. Graphs indicate the sequencing speed of minION devices used in our experiments. Indicated over time in non-cumulative (left) and cumulative (right) bins, are the number of sequenced bases, number of sequenced reads, the number of CpG methylation calls (independent of relevance to 450K arrays) and read length. Last plot indicates the number of CpG methylation calls versus the number of sequenced bases. Blue colors are R9 chemistry, flowcells and workflow and red hues are R10 chemistry, flowcells and workflow.


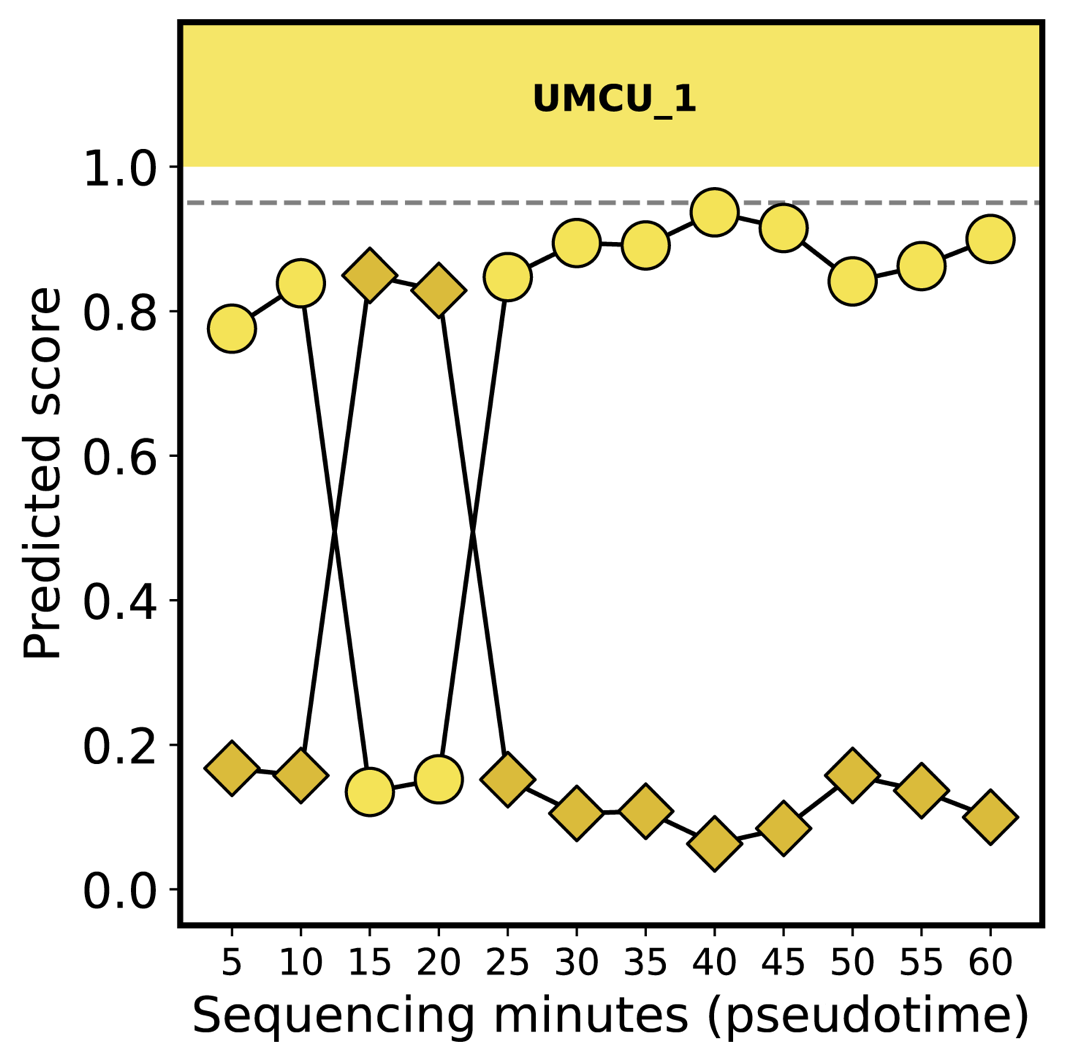


**Supplementary Figure 13:** Classification results from a retrospective oligodendroglioma case (UMCU_1). Sequencing results in high but <0.95 confidence scores for IDH mutant Astrocytoma (dark yellow) and IDH mutant oligodendroglioma (light yellow)

**Supplementary Figure 14:** Copy Number Variation profiles for PMC_60. Sample was sequenced on a nanopore minION to a depth of ~350.000 sequence reads. Top graph shows the CNV profile for the full dataset. The second plot shows the segmentations obtained from the full profile in red, blue dashed lines indicate the segments found in 10 independent samplings of 50.000 and 20.000 sequence reads. The bottom plot shows the CNV profile as it was generated from Whole Exome Sequencing.

**Supplementary Figure 15:** Copy Number Variation profiles. Copy Number Variations shown for nanopore sequenced samples PMC_29, PMC_69, PMC_53 and PMC_42. Sample was sequenced on a nanopore minION, depth is shown in the legend. Top graph shows the CNV profile as obtained from Whole Exome Sequencing. The second plot shows the segmentations obtained from the full profile in red, blue dashed lines indicate the segments found in 10 independent samplings of 50.000 sequence reads.

**Supplementary Figure 16:** Brainstem classifier confusion matrix and F1 scores. Sturgeon brainstem performance on the four test folds of the Capper et al. dataset. Confusion matrix showing the highest scoring class for each reference label at 40 minutes of simulated sequencing (~97% missing values from microarray data). Bars on the right side of the plot indicate the top 1 (solid) and top 3 (transparent) F1-scores per reference label class.


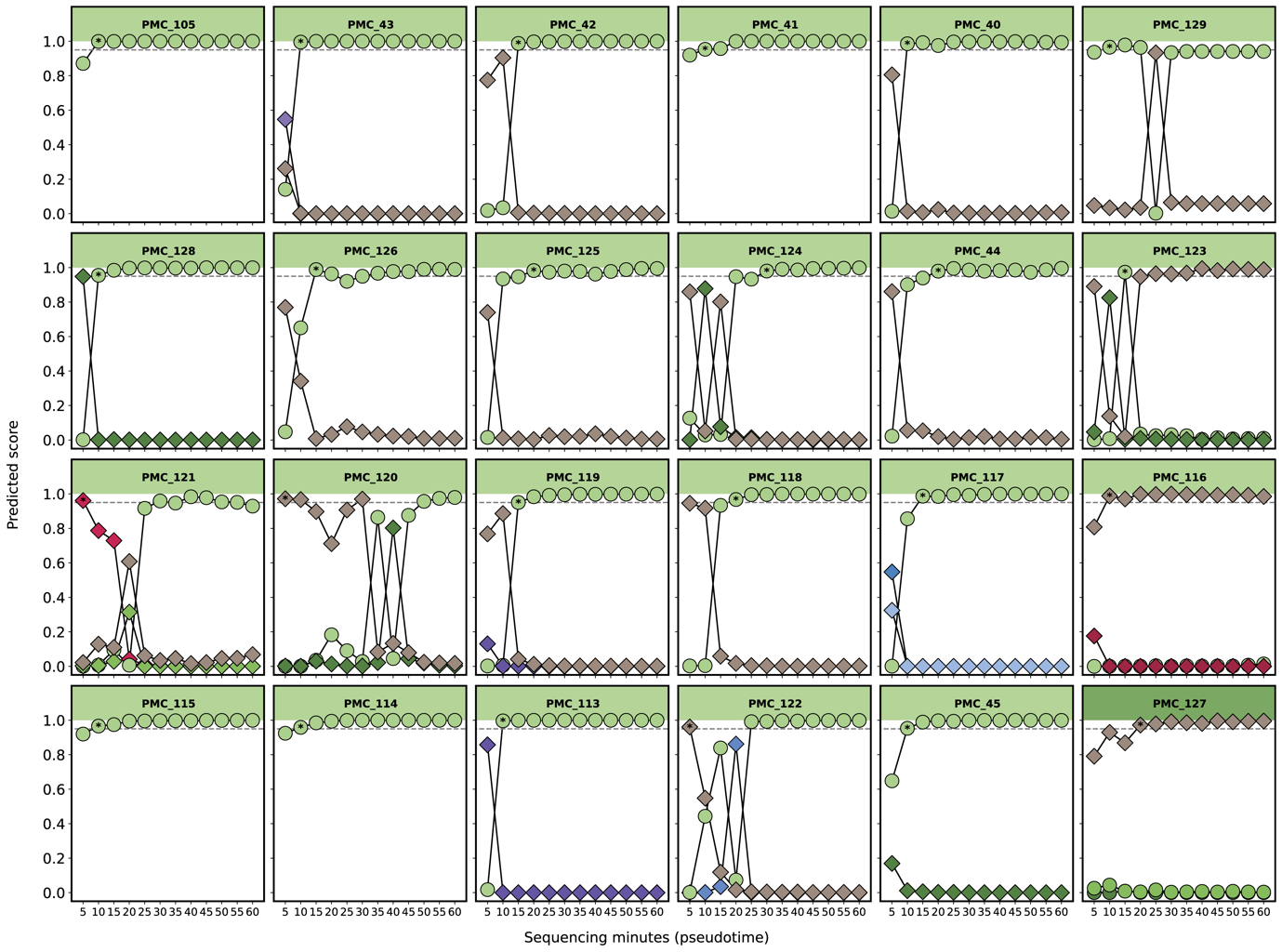


**Supplementary Figure 17:** Confidence over time for the brainstem classifier on brainstem samples. These plots show the confidence of the brainstem classifier with reads accumulated at a rate expected for a minION run. Asterisks indicate the first timepoint the confidence score is higher than 0.95. Brown colors indicate that the model classifies a sample as "non-brainstem".


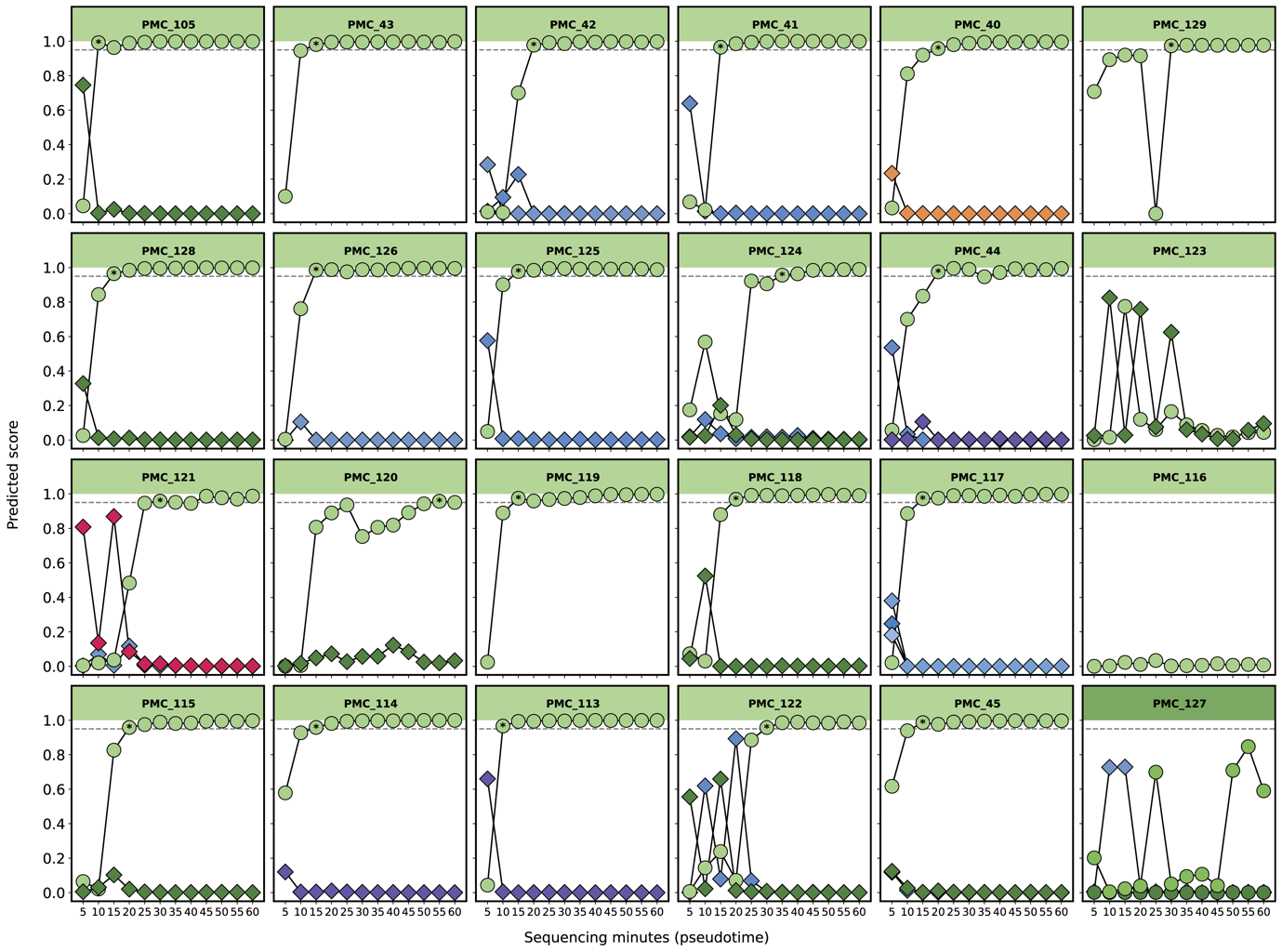


**Supplementary Figure 18:** Confidence over time for the general classifier on brainstem sam- ples. These plots show the confidence of the general classifier with reads accumulated at a rate expected for a minION run. Asterisks indicate the first timepoint the classification score is higher than 0.95.


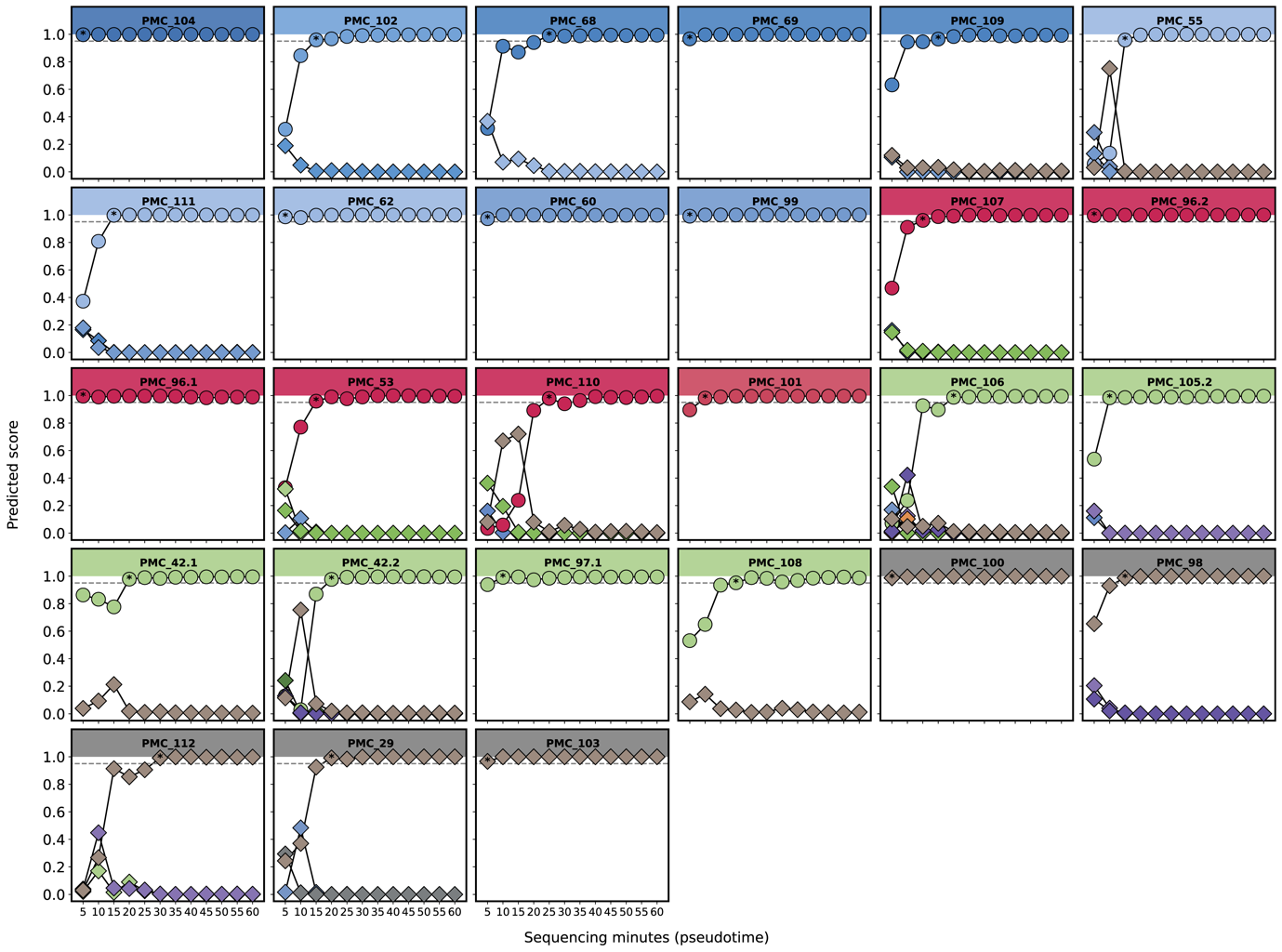


**Supplementary Figure 19:** Confidence over time for the brainstem classifier on non-brainstem samples. These plots show the confidence of the brainstem classifier with reads accumulated at a rate expected for a minION run. Asterisks indicate the first timepoint the classification score is higher than 0.95. Grey samples are from classes not present in the brainstem classifier. Brown colors indicate that the model classifies samples as non-brainstem.


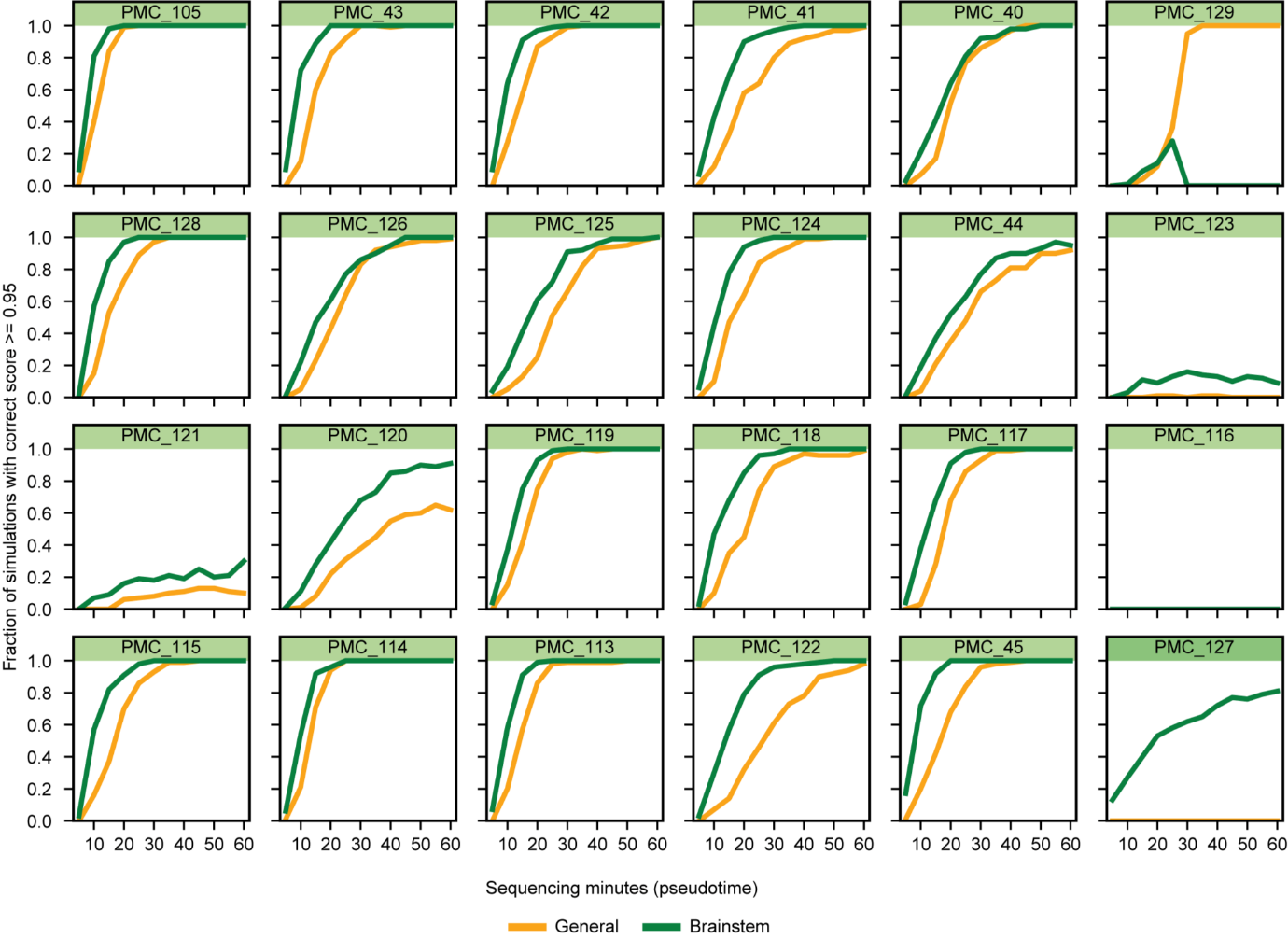


**Supplementary Data Figure 20:** Robustness of the brainstem and general classifier for brain- stem samples. Reads were randomly sampled for each timepoint 100x and classified by the brain- stem and general classifier. Lines indicate the fraction of simulations that reached a >0.95 confidence score for the correct class in the general (orange) and brainstem (green) classifier.


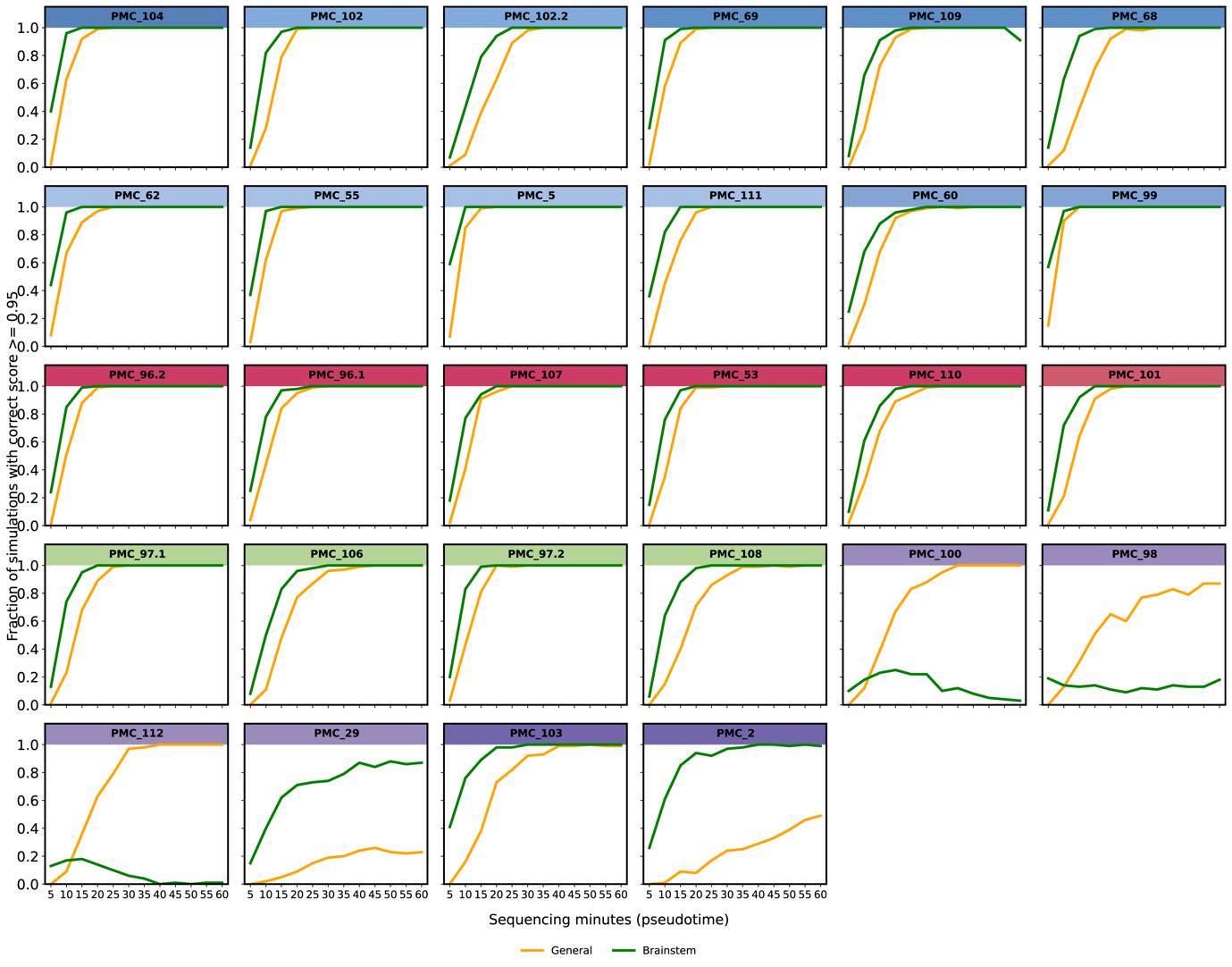


**Supplementary Figure 21:** Robustness of the brainstem and general classifier for non-brain- stem samples. Reads were randomly sampled for each timepoint 100x and classified by the brainstem and general classifier. Lines indicate the fraction of simulations that reached a >0.95 confidence score for the correct class in the general (orange) and brainstem (green) classifier.


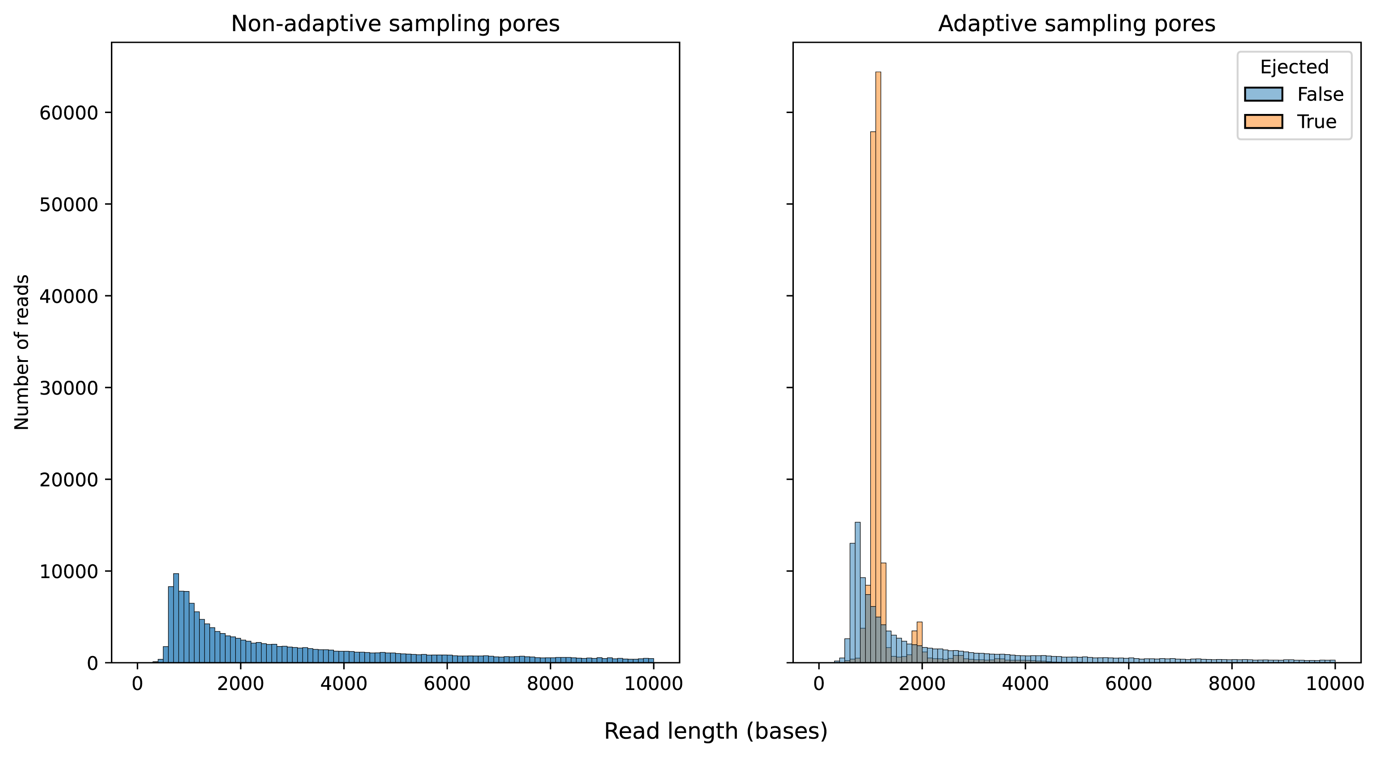


**Supplementary Figure 22:** Read length adaptive versus non adaptive sampling. For a single sequence experiment with adaptive sampling enabled on half of the channels, the left plot shows the read length distribution for non-adaptive sampling channels. The right plot shows the read length distribution for the adaptive sampling channels, with lengths colored by whether the read was rejected or not.


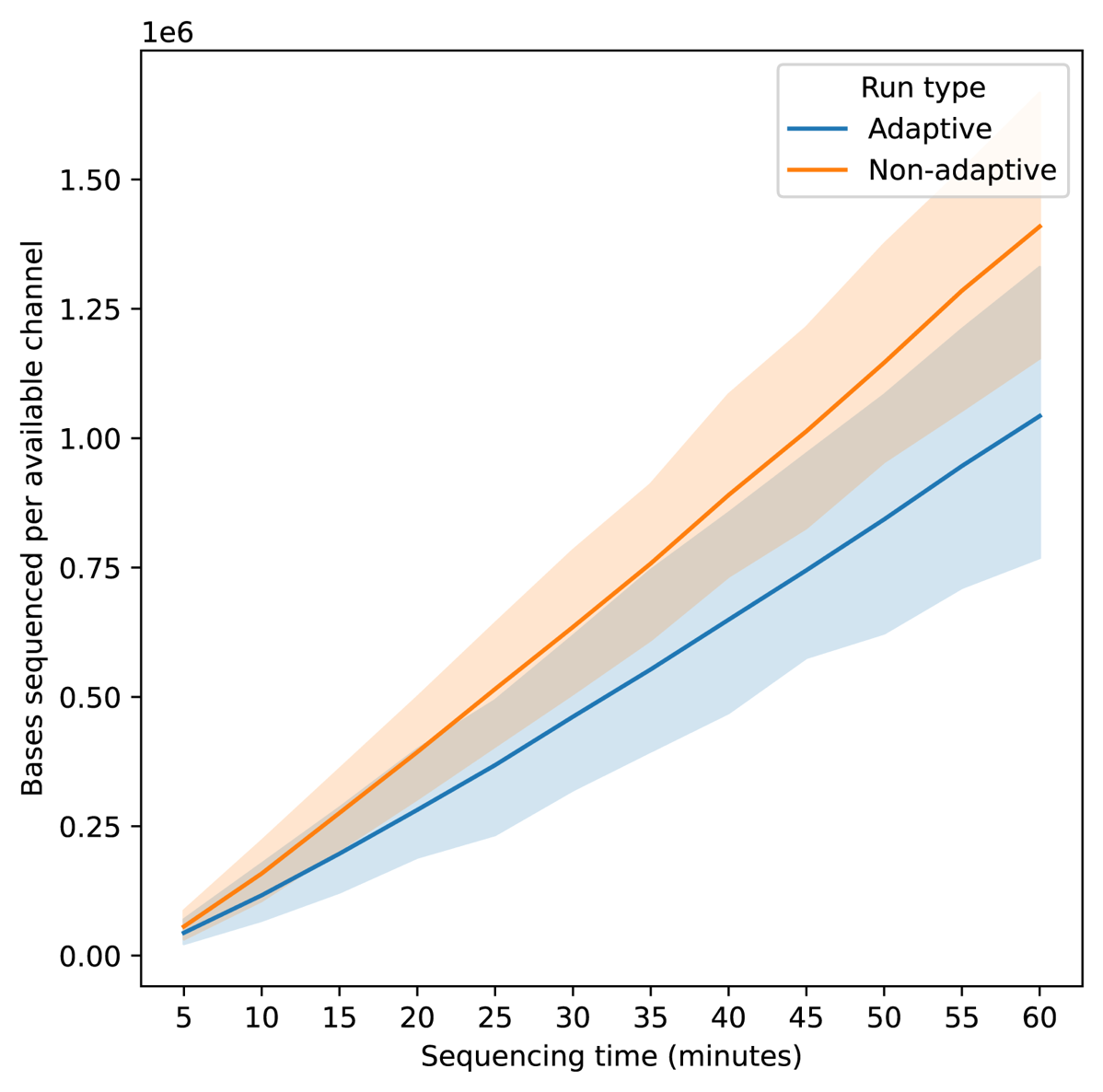


**Supplementary Figure 23:** Throughput of adaptive versus non adaptive channels. For both adaptive and non-adaptive channels, the number of sequenced bases was calculated over time. As expected, adaptive channels spend time ejecting reads, and thus lose some throughput. Solid line indicates the mean, the shadowed area indicates the standard deviation (n=5)


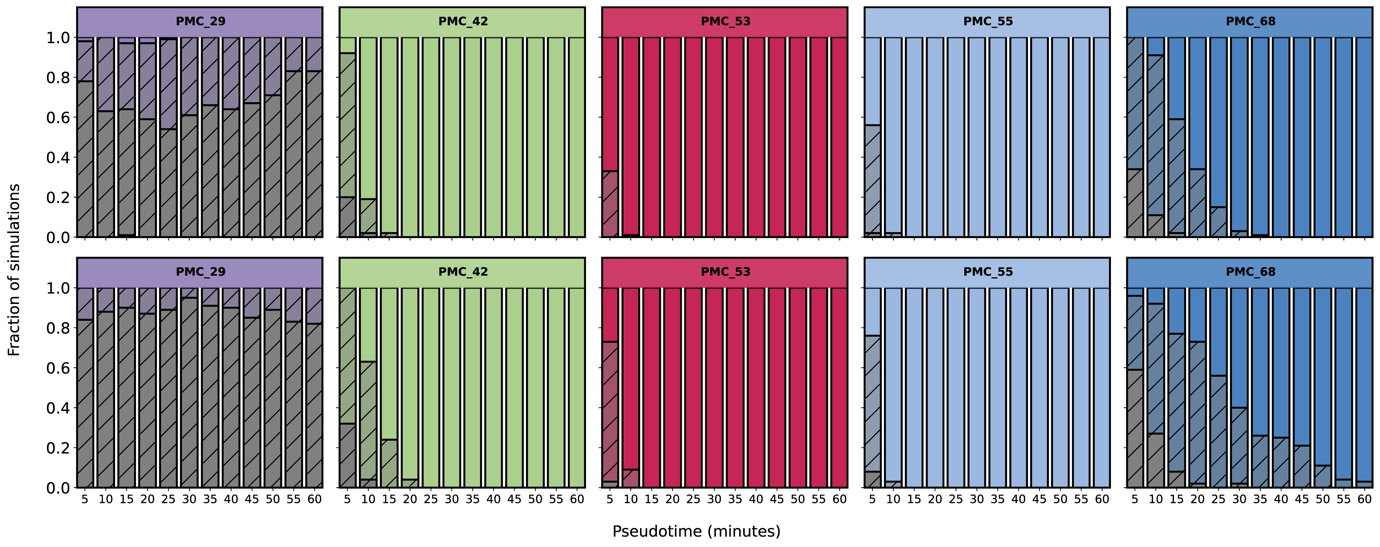


**Supplementary Figure 24:** Robustness of adaptive versus regular sequencing. Five samples were sequenced using adaptive sampling enabled on half of the channels. The top row shows the results of 100 simulations for each of 12 different simulated sequencing times. The fraction of correct classifications is shown in the class color, dashed lines indicate the number of classifications with a score <0.95. Gray dashed lines indicate an incorrect class had the highest confidence score but <0.95, black indicates simulations with a wrong class and confidence >0.95 (N=1).
