# Supplementary material for: Ultra-fast deep-learned CNS tumour classification during surgery
Source: Nature. 2023 Oct 11;622(7984):842–9. doi: 10.1038/s41586-023-06615-2 (PMC10600004; doi:10.1038/s41586-023-06615-2)
Supplement: Supplementary file 2 — Reporting Summary [file 41586_2023_6615_MOESM2_ESM.pdf]

nature portfolio

Corresponding author(s): Jeroen de Ridder

Last updated by author(s): Nov 8, 2023

Reporting Summary

Nature Portfolio wishes to improve the reproducibility of the work that we publish. This form provides structure for consistency and transparency in reporting. For further information on Nature Portfolio policies, see our Editorial Policies and the Editorial Policy Checklist.

Statistics

For all statistical analyses, confirm that the following items are present in the figure legend, table legend, main text, or Methods section.

n/a	Confirmed	
☐	☒	The exact sample size (*n*) for each experimental group/condition, given as a discrete number and unit of measurement
☒	☐	A statement on whether measurements were taken from distinct samples or whether the same sample was measured repeatedly
☒	☐	The statistical test(s) used AND whether they are one- or two-sided<br>*Only common tests should be described solely by name; describe more complex techniques in the Methods section.*
☒	☐	A description of all covariates tested
☒	☐	A description of any assumptions or corrections, such as tests of normality and adjustment for multiple comparisons
☐	☒	A full description of the statistical parameters including central tendency (e.g. means) or other basic estimates (e.g. regression coefficient) AND variation (e.g. standard deviation) or associated estimates of uncertainty (e.g. confidence intervals)
☒	☐	For null hypothesis testing, the test statistic (e.g. *F*, *t*, *r*) with confidence intervals, effect sizes, degrees of freedom and *P* value noted<br>*Give P values as exact values whenever suitable.*
☒	☐	For Bayesian analysis, information on the choice of priors and Markov chain Monte Carlo settings
☒	☐	For hierarchical and complex designs, identification of the appropriate level for tests and full reporting of outcomes
☒	☐	Estimates of effect sizes (e.g. Cohen's *d*, Pearson's *r*), indicating how they were calculated

Our web collection on statistics for biologists contains articles on many of the points above.

Software and code

Policy information about availability of computer code

Data collection	MinKNOW v22.10.10, v22.12.7, v22.12.5
Data analysis	ONT Software: Guppy 5.0.11 and v6.3.8, Megalodon 2.5.0., qCat v1.1.0. Code languages: R 4.1.2, Python 3.7.8. R Packages: Rhdf5 2.38.1., QDNAseq v1.30.0, DNAcopy v1.68.0. Python packages: torch v1.10.2, numba v0.56.2, onnxruntime v1.12.1, scipy v1.7.3, scikit-learn v1.0.2, pandas v1.3.5, numpy v1.21.5, matplotlib v3.5.1, pysam v0.19.0, modbampy v0.6.3. Standalone software: Bedtools v2.30, samtools v1.13

For manuscripts utilizing custom algorithms or software that are central to the research but not yet described in published literature, software must be made available to editors and reviewers. We strongly encourage code deposition in a community repository (e.g. GitHub). See the Nature Portfolio guidelines for submitting code & software for further information.

Data

Policy information about availability of data

All manuscripts must include a data availability statement. This statement should provide the following information, where applicable:

- Accession codes, unique identifiers, or web links for publicly available datasets
- A description of any restrictions on data availability
- For clinical datasets or third party data, please ensure that the statement adheres to our policy

Pediatric patient data is available through the European Genome-Phenome Archive EGAS00001007475, data from AUMC patients is accessible through NCBI Gene

Expression Omnibus (GEO) with accession code GSE237874. Data used for training is available at GEO, under accession number GSE109381.

Human research participants

Policy information about studies involving human research participants and Sex and Gender in Research.

Reporting on sex and gender	We refrain from reporting age and sex per individual, as the low number of patients in the Netherlands, and the high diversity of cases could allow identification of individuals based on these parameters.
Population characteristics	Data and tissue of the patients of the Princess Máxima Center used in this study were not selected for specific biological criteria. The samples used in this study should therefore represent a cross-section of the overall population of pediatric CNS tumor patients in the Netherlands. Furthermore we do not compare groups of patients and we do not stratify the patient population in any way, and therefore do not see the need to publish these characteristics.<br>However in summary the group of pediatric patients sequenced consists of 35 male and 33 female patients, with age at diagnosis ranging from 0.3 to 16 years (7.2 years on average). These characteristics are available at the repository should other researchers need these for their work.
Recruitment	Parents and/or patients (depending on the age) in the Princess Máxima Center signed an informed consent for use of their data and tissue for research purposes (opt-in procedure). For Glioma patients from the Amsterdam University Medical center a broad consent was signed for use of material to improve clinical methods. For AUMC patients we publicly share methylation calls, but not raw DNA sequence data as there is no explicit consent for sharing genetic information. Pediatric patients were included on the basis that they underwent resection surgery for a CNS tumor, and adult patients were selected on the basis that they underwent (suspected) Glioma resection surgery.
Ethics oversight	The research was approved by the Biobank and Data access committee (BDAC) of the Princess Máxima Center for pediatric oncology. All included patients provided written informed consent for participation in the biobank (International Clinical Trials Registry Platform: NL7744, https://onderzoekmetmensen.nl/en/trial/21619)). Inclusion of intraoperative samples was ethically approved via the same decision; the results were not shared with caregivers and therefore not used to alter patient treatment nor diagnosis.

Note that full information on the approval of the study protocol must also be provided in the manuscript.

Field-specific reporting

Please select the one below that is the best fit for your research. If you are not sure, read the appropriate sections before making your selection.

☒ Life sciences ☐ Behavioural & social sciences ☐ Ecological, evolutionary & environmental sciences

For a reference copy of the document with all sections, see nature.com/documents/nr-reporting-summary-flat.pdf

Life sciences study design

All studies must disclose on these points even when the disclosure is negative.

Sample size	94 EPIC profiles, 50 retrospective Nanopore sequencing samples and 25 intraoperative Nanopore sequencing samples are included in this study. Sample sizes were determined based on availability, the 94 methylation array profiles form a representative cross section of patients, and by simulation of shallow sequence runs this number is upsampled to allow extensive model validation.<br>50 retrospective sequencing cases were generated (and again upsampled) to confirm that the findings on array profiles can be translated to real sequencing.<br>25 intraoperative cases is enough to reveal the major challenges in clinical practice, we do not claim this number is enough to calculate sensitivity and specificity; further clinical validation is pending, however the low incidence of CNS tumors and the high number of different classes prevents collection of extensive cohorts in a timely manner.
Data exclusions	We did not exclude any samples.
Replication	All technical replication attempts were successful, biological replication; ie sequencing from different samples of the same tumor showed some variability, likely due to sample purity; this is adressed in the article.
Randomization	Randomization is not applicable to this study: we do not compare groups of patients, but instead compare the results of our method to the existing methylation array and Heidelberg classifier performed for the same patients.
Blinding	Blinding was not performed in the study, because the results are based on the classification generated by a neural network algorithm. The black-box nature of such algorithms prevents the operator from influencing the outcome.

Reporting for specific materials, systems and methods

We require information from authors about some types of materials, experimental systems and methods used in many studies. Here, indicate whether each material, system or method listed is relevant to your study. If you are not sure if a list item applies to your research, read the appropriate section before selecting a response.

Materials & experimental systems

n/a	Involved in the study
☒	☐ Antibodies
☒	☐ Eukaryotic cell lines
☒	☐ Palaeontology and archaeology
☒	☐ Animals and other organisms
☐	☒ Clinical data
☒	☐ Dual use research of concern

Methods

n/a	Involved in the study
☒	☐ ChIP-seq
☒	☐ Flow cytometry
☒	☐ MRI-based neuroimaging

Clinical data

Policy information about clinical studies

All manuscripts should comply with the ICMJE guidelines for publication of clinical research and a completed CONSORT checklist must be included with all submissions.

Clinical trial registration	This is a biobank study. All included patients provided written informed consent for participation in the biobank (International Clinical Trials Registry Platform: NL7744).
Study protocol	Not applicable
Data collection	Parents and/or patients (depending on the age) in the Princess Máxima Center signed an informed consent for use of their data and tissue for research purposes (opt-in procedure). The research was approved by the Biobank and Data access committee (BDAC) of the Princess Máxima Center for pediatric oncology. Inclusion of intraoperative samples was ethically approved via the same decision; the results were not shared with caregivers and therefore not used to alter patient treatment nor diagnosis. For Glioma patients from the Amsterdam University Medical center a broad consent was signed for use of material to improve clinical methods. For AUMC patients we publicly share methylation calls, but not raw DNA sequence data as there is no explicit consent for sharing genetic information.
Outcomes	Not applicable since this is a biobank study, results were not used to alter treatments.
